# Emergence of invasive *Escherichia coli* pathobionts in gut microbiome promotes cancer stemness via targeting Hippo pathways

**DOI:** 10.1080/19490976.2026.2694795

**Published:** 2026-06-30

**Authors:** Linda Chia-Hui Yu, Shu-Chen Wei, Been-Ren Lin, Yi-Hsuan Li, Yu-Chien Liao, Yu-Wen Peng, Chia-Hsien Lin, Pei-Tzu Hu, Yu-Chen Pai, Liang-Chuan Lai, You-Tzung Chen, Chung-Yen Huang, Yung-Ming Jeng, Yen-Hsuan Ni

**Affiliations:** a Graduate Institute of Physiology, National Taiwan University College of Medicine, Taipei, Taiwan ROC; b Department of Internal Medicine, National Taiwan University Hospital, Taipei, Taiwan ROC; c Department of Surgery, National Taiwan University Hospital, Taipei, Taiwan ROC; d Graduate Institute of Biomedical Electronics and Bioinformatics, National Taiwan University, Taipei, Taiwan ROC; e Graduate Institute of Medical Genomics and Proteomics, National Taiwan University College of Medicine, Taipei, Taiwan ROC; f Department of Pathology, National Taiwan University Hospital, Taipei, Taiwan ROC; g Department of Pediatrics, National Taiwan University Hospital, Taipei, Taiwan ROC

**Keywords:** Colorectal cancer microbiome, tumorigenic *E. coli*, invasive pathobionts, intestinal epithelial cells, experimental pathobiont models, stem cells, tumorsphere, clonogenicity, hereditary cancer, Hippo signaling

## Abstract

Growing evidence suggests a pivotal role of the microbiome in tumorigenesis, extending beyond genetics. Apc(Min/+) mice exhibit reduced tumor load when housed in germ-free conditions. Nevertheless, how genetic factors shape microbiota and how dysbiosis fits into the genetic paradigm of intestinal carcinogenesis remain elusive. Epithelial stemness is regulated by Wnt/Apc/β-catenin pathway, whereas *Apc* mutations and Hippo signaling are associated with tumor growth. Invasive pathobionts emerge from microbiota as a result of epithelial barrier dysfunction. We hypothesize that the emergence of invasive pathobionts and dysbiosis of epithelial microbiota contribute to increased cancer stemness. The epithelial and fecal microbiota are longitudinally monitored in Apc(Min/+) and wild-type littermates born to wild-type surrogate dams. Segregation of epithelial microbiota between Apc(Min/+) and wild-type mice was observed as early as eight weeks after birth, whereas fecal microbiota diverged at 20 weeks of age. Epithelial dysbiosis and barrier defects were observed in Apc(Min/+) mice, characterized by intraepithelial *Escherichia coli* with invasive features. While antibiotic treatment reduced cancer burden, invasive *E. coli* infection promoted tumorsphere formation. Higher expression of *Vgll3* and *Tead4* (Hippo effectors) and *Cd44* (a cancer stemness marker) was observed in bacteria-infected tumorspheres. Mechanistically, bacteria augmented epithelial clonogenicity by enhancing VGLL3/TEAD4-mediated CD44 promoter activity. Invasive *E. coli* genetic signatures were verified in 86% of human colorectal carcinoma specimens, and a positive correlation with TEAD4 expression was observed. In conclusion, *Apc* mutation drives the expansion of invasive pathobionts to promote cancer stemness via a VGLL3/TEAD4/CD44 axis. Bacteria-targeting interventions could be an alternative strategy for patients with hereditary tumors.

## Graphical abstract

**Figure:**
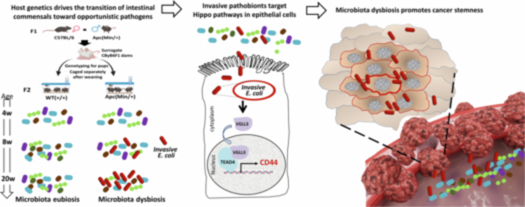

## Introduction

Intestinal cancers primarily originate from epithelial cells that form adenocarcinomas at the interface between host and microbiota. In addition to genetic mutations, it is now widely acknowledged that microbiota dysbiosis plays a crucial role in the development of colorectal carcinoma (CRC). A higher abundance of *Escherichia coli* was reported in fecal and mucosal biopsy specimens from patients with familial adenomatous polyposis (FAP) and CRC than in those from healthy subjects.^1-3^ Individuals with hereditary FAP caused by mutations in the adenomatous polyposis coli (APC) gene develop numerous benign polyps at a young age throughout both small and large intestines, with a higher risk of malignant transformation.^4^ The abundance of mucosa-associated *E. coli* in FAP patients implied that dysbiosis could be a cause rather than a consequence of neoplasms. Nevertheless, the origins of dysbiosis and its role within the genetic paradigm of CRC remain poorly defined.

The causal role of the microbiota in CRC development has been validated through long-term antibiotic treatment and fecal transplantation in experimental models.^5-9^ Mice with an *Apc* gene mutation that spontaneously develop multiple intestinal neoplasia (Min) in conventional housing conditions display an absence of tumors or a low tumor burden when raised in germ-free facilities.^8^ ^,^ ^9^ Conversely, fecal microbiota transfer from CRC patients increased the tumor load in Apc(Min/+) mice compared with stool transfer from healthy individuals.^8^ ^,^ ^9^ It is noteworthy that adenoma-linked barrier defects and bacterial penetration are early events in epithelial cancers driven by *Apc* allelic loss.^10-13^ The link between microbiota and carcinogenesis was further strengthened by findings on commensal-derived opportunistic pathogens, termed pathobionts. We and others demonstrated that adherent-invasive *E. coli* promoted tumor growth by inducing epithelial hyperproliferation, genotoxicity, and cell senescence.^14-19^ The *E. coli* strain LI60C3 accelerates cell-cycle progression and cancer initiation, with higher levels of genetic signatures observed in colorectal cancer samples.^14^ ^,^ ^15^ Other *E. coli* strains, such as NC101, CCR20, and 11G5, produce genotoxins that cause DNA damage in host cells and contribute to tumorigenesis.^16-19^ Recent evidence shows that invasive *E. coli* pathobionts in the gut microbiome emerge due to dysfunction of the epithelial barrier.^20^ ^,^ ^21^ We hypothesize that increased permeability to bacteria and the emergence of invasive pathobionts are involved in tumor growth in Apc(Min/+) mice. While the commensal roots of *E. coli* pathobionts are generally accepted, longitudinal data tracing the conversion of resident microbes from eubiotic commensals to invasive bacteria are still lacking.

Polymorphic microbiomes and dysregulated stemness (self-renewal properties) are two characteristics that have recently been added to the hallmarks of cancer.^22^ Whether *E. coli* pathobionts trigger cancer stemness remains unclear. Unlike stem cells in the intestinal crypts, which maintain epithelial homeostasis, cancer stem cells (cancer-initiating cells) undergo symmetrical cell division to achieve high clonogenicity and hyperproliferation. Stem cell proliferation is primarily regulated by the Wnt/β-catenin pathway, in which Lgr5 interacts with the Wnt receptor complex. Similar to a constitutively active Wnt signal, *Apc* gene mutation leads to persistent nuclear translocation of β-catenin, thereby increasing transcription factor 4-dependent cell cycle gene expression.^23^ Aside from Wnt/β-catenin signals, the Hippo pathway is linked to stemness characteristics in colorectal carcinoma, head and neck squamous carcinoma, and ovarian, breast, and hepatic carcinomas.^24-26^ It comprises TEAD transcriptional activity, facilitated by the nuclear translocation of cofactors such as Yes-associated protein (YAP), transcriptional coactivator with PDZ-binding motif (TAZ), or Vestigial-like family member 3 (VGLL3).^26-29^ Cancer stem cells that highly expressed CD44 and CD133 markers exhibit increased clonogenicity *in vitro* and *in vivo*.^30-32^ In addition to the standard form of CD44 (CD44s), splicing variants (v) 3 and 6 are associated with poor tumor prognosis. Recent studies have shown that TEAD4 acts as a novel transcriptional factor that binds the CD44 promoter and modulates CD44 isoform expression in adenocarcinomas.^33^ ^,^ ^34^

This study explored changes over time in both the epithelial and fecal microbiota and characterized intraepithelial bacteria in Apc(Min/+) mice. The impact of invasive *E. coli* on cancer growth and tumor clonogenicity was evaluated in mouse models, as well as *in vitro* organoid cultures and human epithelial cell lines. Additionally, the study investigated the signaling pathways and transcriptional activities underlying *E. coli*-induced cancer stemness. The correlation between microbial signatures and host gene expression was validated in human CRC specimens.

## Materials and methods

### Ethical approval for studies

All experimental procedures were approved by the Institute of Animal Care and Use Committee (IACUC) (#20210026 and #20230363) of NTUCM. Approval of human sample collection was granted by the Institutional Review Board (IRB) of NTUH (202104001RINC and 202405004RINB).

### Animal housing conditions

All mice were housed in temperature-controlled rooms (23 °C ± 2 °C) with 12-h light-dark cycles in the specific pathogen-free (SPF) animal facility at NTUCM. Mice were fed standard chow and water *ad libitum*.

### Genetic mutant Apc(Min/+) mouse model

Mice with a single allele of the *Apc* gene mutation bearing multiple intestinal neoplasia (Apc(Min/+)) were generated through aseptic *in vitro* fertilization (IVF) by using C57BL/6 oocytes and Apc(Min/+) sperm. The resulting embryos were then implanted in the wombs of surrogate CByB6F1 dams (a cross-strain between C57BL/6 and BALB/c mice).^35^ The surrogate wild-type dams that carried the embryos to term were housed in SPF conditions. The offspring, comprising the Apc(Min/+) and WT(+/+) littermates in a 1:1 ratio, were nursed by the surrogate dam until weaning at 4 weeks of age. The littermates were genotyped and separated by cage after weaning, and they were kept until 20 weeks of age. Our previous breeding strategy, which involved intercrossing of Apc(Min/+) males with C57BL/6 females, resulted in high offspring mortality. Therefore, the IVF strategy was used to generate all batches of mice for experiments.

### Antibiotic treatment in mice

The Apc(Min/+) mice were randomly divided into four experimental groups (*N* = 18–20/group). Antibiotic (ABX) mixtures (vancomycin 500 mg/L, neomycin 1 g/L, metronidazole 1 g/L, and ampicillin 1 g/L) were administered to Apc(Min/+) mice via daily oral gavage and drinking water supplementation.^14^ ^,^ ^36^ The mice were treated with ABX at 6, 8, 10, or 12 weeks of age for one week, then returned to normal water until 20 weeks of age for tumor burden measurement.

### Experimental pathobiont models

The Apc(Min/+) mice were randomly divided into three experimental groups at 8 weeks of age (*N* = 18–20/group). The mice received a one-week ABX treatment followed by oral administration of LI60C3 (an adherent-invasive *E. coli* strain) or *htrA* gene-deleted bacteria (ΔHtrA) at 10^9^ colony-forming units (CFUs) per mouse, administered in three consecutive doses every other day. The vehicle control group received phosphate-buffered saline (PBS) orally at the same time points as the pathobiont groups, with all groups beginning 1 d after ABX withdrawal. The disturbance of microbiota by ABX treatment facilitates colonization of the *E. coli* pathobiont in the intestine. All mice were kept in cages under SPF conditions until 20 weeks of age to determine tumor burden. Paraffin-embedded intestinal sections were used for histopathological examination and tumor grading by a blinded pathologist.^37^ ^,^ ^38^

In addition, pathobiont infection was performed in a chemically induced cancer model. Specific pathogen-free BALB/c mice (6–8 weeks of age) (*N* = 10/group) were intraperitoneally injected with azoxymethane (AOM) (10 mg/kg body weight), and after 7 d, given 2% dextran sodium sulfate (DSS) in drinking water for 4 d, followed by 3 d of normal water. Mice were subjected to three cycles of AOM/DSS and administered one week of ABX mixtures on days 56–63, followed by oral administration of bacteria (10^9^ CFUs) on day 64, and tumors were assessed on day 90.^37^ ^,^ ^38^

### Microbiota 16S rDNA sequencing

Epithelial and fecal samples were collected at 4, 8, and 20 weeks of age in Apc(Min/+) mice. The samples were extracted for bacterial DNA using a QIAamp DNA stool mini kit (Qiagen), and subjected to high-throughput full-length 16S rDNA sequencing on the PacBio Sequel II platform. Microbial operational taxonomic units (OTUs) were used in alpha rarefaction analysis to estimate species richness with the Shannon method. The beta rarefaction analysis, conducted using the Bray-Curtis weighted Unifrac method, determines the diversity between samples. The Principal Coordinates Analysis (PCoA) was also used to assess differences in microbial community structure across groups.^14^ ^,^ ^20^ ^,^ ^39^ The bacterial 16S rDNA gene sequencing data were submitted to the GEO database (#GSE245618).

### Intestinal epithelial cell isolation

Intestinal segments were excised and flushed with ice-cold PBS for epithelial cell isolation following an established dithiothreitol (DTT)-ethylenediaminetetraacetic acid (EDTA) method.^20^ ^,^ ^21^ The epithelial cells were used for a gentamicin resistance assay and RNA extraction for further analysis.

### Gentamicin resistance assay for intraepithelial bacterial counts

The isolated epithelial cells were incubated with 300 μg/mL gentamicin (Sigma) for 1 h, lysed with 1% Triton X (TX)-100 in PBS for 10 min, and plated on blood agar plates overnight.^36^ Gentamicin is an antibiotic that is bactericidal against extracellular microbes but leaves intracellular bacteria intact. The number of bacterial colonies is normalized to that of trypan blue-negative epithelial cells, and presented as log_10_ CFU per 10^6^ cells. Genomic DNA was also extracted from single bacterial colonies and sent for sequencing and classification.

### Bacterial culturing

The *E. coli* strain LI60C3 was isolated from intestinal epithelial cells of a mouse model of colitis-associated cancers, and *htrA* gene-deleted bacteria were engineered using the phage *λ*-red and Flp recombinase method.^14^ ^,^ ^15^ ^,^ ^20^ Moreover, a plasmid pRSET containing green fluorescent protein (GFP) was transformed into LI60C3.^21^ Other *E. coli* strains were obtained from the ileal or colonic epithelial cells of Apc(Min/+) mice, including A748-I1, A727-I1, A743-I1, A844-I1, A727-C1, A775-C1, A837-C1, and A844-C1. A laboratory K-12 sub-strain of *E. coli* (DH5α) derived from human feces was used for comparison, which is known to be nonpathogenic and noninvasive. On the day of the experiments, a single bacterial colony was inoculated into Luria-Bertani broth and harvested at mid-exponential phase (OD600 of 0.6–0.8), and the bacterial pellets were resuspended in PBS for further experiments.

### Primary mouse intestinal organoid cultures

Intestinal crypts were collected for primary culturing 2 d after bacterial inoculation. Briefly, cell clusters of the crypt fractions were isolated by using an EDTA-detachment method, and 1500 cell clusters were plated in Matrigel (BD Biosciences, #356235) at a ratio of 3:1 with crypt culture media and then overlaid with media. The organoids were incubated for up to 6 d, and a total of 180-200 organoids were quantified per mouse group by image capturing.^37^ ^,^ ^38^

### Western blotting

The proteins in mouse tissues and human cell cultures were extracted with a radioimmunoprecipitation assay (RIPA) buffer containing a tablet of Complete-Mini (C-M, a protease inhibitor cocktail) (Roche) on ice for 1 h, and the protein concentration was normalized using a Dual-Range BCA Protein Assay Kit (Visual Protein, Taiwan). For fractionation of cellular components, the cells were first extracted with a 0.6% NP-40 solution containing C-M to obtain cytoplasmic fractions. The nuclear pellets were obtained after centrifugation at 16000 × *g* for 15 min once and 3 min thrice, and then sonicated. The total protein samples were adjusted to 2 mg/ml, and the cytoplasmic and nuclear protein samples were adjusted to 1 mg/ml. The samples were then subjected to gel electrophoresis (4%–13% polyacrylamide), and transferred to PVDF membranes for incubation with the antibodies stated below, and detected by chemiluminescent signals.^37^ ^,^ ^38^

The primary antibodies included monoclonal mouse anti-human TEAD4 (Santa Cruz, Cat# sc-390578), polyclonal rabbit anti-human YAP (1:1000, Cell Signaling, Cat# 4912), monoclonal rabbit anti-mouse TAZ (1:1000, GeneTex, Cat# GTX637933), mouse anti-human VGLL3 (1:1000, Abcam, Cat# ab68262), monoclonal rabbit anti-human CD44v6 (1:500, Invitrogen, Cat# 701406), monoclonal rabbit anti-human β-catenin (1:5000, Abcam, Cat# ab32572), monoclonal mouse anti-α-tubulin (1:1000, Santa Cruz, Cat# sc-5286), monoclonal mouse anti-human Lamin A (1:1000, Santa Cruz, Cat#sc-7292), and monoclonal mouse anti-human β-actin (1:10000, Sigma, Cat# A5411). The secondary antibodies were horseradish peroxidase-linked goat anti-rabbit IgG or horse anti-mouse IgG (1:2000, Cell Signaling, Cat# 7074 and 7076).

### Human cell lines for bacterial challenge

Human colorectal adenocarcinoma Caco-2 cell lines were grown in Dulbecco's Modified Eagle's Medium (DMEM) as previously described and used for bacteria-epithelial cocultures.^21^ ^,^ ^40^ The cells were pretreated with 2 µM verteporfin (a specific inhibitor of TEAD activation, Sigma-Aldrich, Cat# 12764) prior to bacterial exposure.^33^ ^,^ ^41^ ^,^ ^42^ The cells were apically exposed to bacteria in antibiotic-free culture medium at a multiplicity-of-infection (MOI) of 1 or 10, and incubated at 37 °C for 4 h. The epithelial monolayer was rinsed twice with sterile PBS and incubated with 1 mM EDTA to obtain single cells for quantifying intracellular bacterial counts by using a gentamicin resistance assay.^36^

In some experiments, epithelial cells were processed for cytoplasmic and nuclear fractionation, and protein levels were measured by Western blotting immediately after a 4-h bacterial incubation. In other settings, after washing off the extracellular bacteria, the epithelial cells were incubated in culture media containing gentamicin (300 μg/ml) for 24 h to evaluate cell cycle progression by flow cytometric analysis, and for 24-96 h to assess total protein and gene expression by Western blots and qPCR analysis.^37^ ^,^ ^38^

### Measurement of stemness by clonogenic assays

Clonogenic assay is a widely used *in vitro* method to quantify self-renewing cells, based on the ability of a single cell to grow into a colony.^43^ ^,^ ^44^ Our protocol for Caco-2 cells was modified from the assay developed by Franken et al.^43^ ^,^ ^44^ Confluent Caco-2 cell monolayers grown in 12-well plates were apically exposed to bacteria at an MOI of 10 for 4 h in an antibiotic-free culture medium. After rinsing with PBS twice to remove extracellular bacteria, the cells were incubated in culture media containing gentamicin (300 μg/ml) for 6 d. The bacteria-exposed Caco-2 cells were then trypsinized and resuspended as single cells in fresh gentamicin-containing culture media. The single cells were reseeded into 6-well plates (a surface area of 9.6 cm² per well) at a concentration of 100-150 cells/ml in 2  ml of culture medium and incubated for an additional 10 d. At the end of the experiments, the Caco-2 cells were fixed with 4% paraformaldehyde for 15 min and stained with 0.1% crystal violet for 15 min. The number and area of Caco-2 cell colonies, defined as containing at least 50 cells, were measured using ImageJ 1.47v software. The clonogenic efficiency was determined as the number of colonies divided by the total number of cells at reseeding.^44^

### Spheroid cultures

The monolayered Caco-2 cells exposed to bacteria for 4 h were trypsinized and resuspended in culture medium containing gentamicin (300 μg/ml) to eliminate extracellular bacteria, and reseeded into spheroid cultures. The cells were immediately mixed with ice-cold Matrigel (Corning #354234) at a 3:1 ratio with cell culture medium. After gel polymerization, a gentamicin-containing culture medium was overlaid onto the cells and replaced every 2 d. Spheroids were cultured for 6 d, and the images were captured using a CCD camera. The area of each spheroid from a total of 180-200 spheroids was analyzed using ImageJ software.^33^ ^,^ ^45^

### Immunofluorescent staining in cell cultures

Cell monolayers exposed to fluorescent *E. coli*-GFP were processed for immunofluorescent staining. The cells on filter supports were fixed with 4% paraformaldehyde, permeabilized with 0.1% TX-100, and quenched with 50 mM NH_4_Cl. After blocking with 1% BSA for 2 h, cells were stained with primary antibodies, rabbit polyclonal anti-ZO-1 (1:100, Thermo Fisher Scientific, Cat# 40-2200), followed by a secondary antibody, goat anti-rabbit IgG conjugated to Alexa Fluor 594 (1:1000, Thermo Fisher Scientific, Cat# A-11012). The cell nuclei were counterstained with a Hoechst dye (1 μg/ml) in PBS. The fluorescent images were captured using a Zeiss microscope.^46-48^

### Flow cytometric analysis of cell cycle rates

The bacteria-exposed epithelial cells were isolated to single cells and fixed with ethanol, followed by incubation with a primary antibody rabbit anti-human Ki67 (1:400, Cell Signaling, Cat# 9129), and a secondary antibody Alexa Fluor 488-conjugated goat anti-rabbit IgG (Thermo Fisher Scientific, Cat# A-11034), and stained with propidium iodide (PI) (Sigma-Aldrich, Cat# P4170). A minimum of 10,000 PI-stained nuclei were analyzed by flow cytometry for cell cycle phases using Mod Fit LT cell cycle analysis software.^37^ ^,^ ^38^

### Plasmid constructs and cell transfection

Cells were transfected with plasmids, including pcDNA3.1 (Addgene_79663) carrying TEAD4, YAP1, TAZ, and VGLL3 genes (2.5 μg/well each) or mock plasmids by lipofection, and the transfection efficiency was confirmed by PCR analysis.

### Lentiviral delivery of shRNA for gene silencing

The lentivirus was assembled from shRNA oligonucleotides targeting the *TEAD4* gene in a modified pLKO.1-puromycin vector, packaging plasmids (pCMV-R8.74psPAX2), and envelope plasmids (pMD2.G), which were constructed in the RNA Technology Platform and Gene Manipulation Core in Academia Sinica (Taipei, Taiwan). Briefly, lentiviral media produced from HEK293T cells were filtered through a 0.45-µm pore size filter and stored until use. For the gene knockdown experiment, Caco-2 cells at 50% confluency were infected with lentiviral media mixed with culture media at a 1:1 ratio, with 8 µg/mL polybrene, for 2 d. The cells were selected by culturing in media containing 8 µg/mL puromycin for 2 d, then challenged with bacteria at an MOI of 10 for 4 h, followed by spheroid culture and flow cytometric analysis.^20^ ^,^ ^46^

### Luciferase-driven CD44 promoter activity assay in Caco-2 cells

The expression vector pcDNA3.1 carrying the target gene sequence and the luciferase reporter vector pGL3-Basic vector with the human CD44 promoter were constructed in the Biomedical Resource Core in NTUCM. For the luciferase reporter assay, Caco-2 cells were seeded into 24-well plates at a concentration of 2 × 10^5^ cells per well for 16 h. The cells were transfected with pcDNA3.1 carrying TEAD4, YAP1, TAZ, or VGLL3, and with pGL3-CD44 promoter-Luc, and pGL4.74-hRluc by using jetPRIME® Transfection reagents. In some settings, the cells were placed in a 37 °C incubator for transfection for 48 h and then challenged with bacteria at MOI =  10 for 4 h and the cell media was replaced with gentamicin-containing media for incubation of 48 h. The cells were then lysed and the resultant supernatant was analyzed using a Dual-Glo Luciferase assay (Promega).^21^ ^,^ ^33^

### Polymerase chain reaction (PCR)

The extracted RNA was reverse-transcribed with random hexamer primer or oligo(dT)_15_using the RevertAid First Strand cDNA Synthesis kit (Thermo Scientific), and quantitative PCR was performed using a StepOnePlus Real-Time PCR System (Applied Biosystems) as described.^14^ ^,^ ^21^ ^,^ ^48^ Each sample was run in duplicate, and the mean threshold cycle (CT) was determined from the two runs. The CT value of the target gene was subtracted from that of the housekeeping gene (ΔCT), and the double delta CT value (ΔΔCT) was determined as the difference between the ΔCT values of the treatment and control groups. The relative gene expression was calculated as the logarithm of the value of −ΔΔCt to base 2 (2^−^^ΔΔCT^). All primer pairs were designed in our laboratory based on NCBI database gene sequence (Suppl Tables 1 and 2).

### Surgical specimens from CRC patients

Patients with CRC at four tumor stages were recruited as subjects at National Taiwan University Hospital (NTUH). Written informed consent was obtained from all study subjects, and approval for this study was granted by the Institutional Review Board (IRB) for NTUH (202104001RINC, 202405004RINB). The tumor tissues were extracted separately for RNA and DNA contents, with the reverse-transcribed cDNA samples used for human gene expression analysis and the genomic DNA samples for bacterial gene quantification (Suppl Table 3). The Pearson correlation coefficient (*r*), which measures the strength and direction of a linear relationship between the two variables, was then calculated for the bacterial and host gene levels.

### Bioinformatics analysis

The RNA Seq-based gene expression datasets were generated by The Cancer Genome Atlas Program (TCGA) Research Network (http://cancergenome.nih.gov). The TCGA datasets were analyzed using computational programs of cBioPortal (https://www.cbioportal.org) and University of California Santa Cruz (UCSC) Xena (https://xenabrowser.net).

### Statistical analysis

Data are presented as mean ± SEM, unless otherwise stated. The data that fit the normality assumption were analyzed using ANOVA followed by Tukey's or Dunnett's post hoc tests for comparing multiple groups and Student's *t*-test for comparing two groups. Nonparametric analysis was also employed when the data failed the normality test. A *p* value less than 0.05 is considered significant.

## Results

### Microbiota are involved in the tumorigenic mechanisms of Apc(Min/+) mice

**Figure 1.**
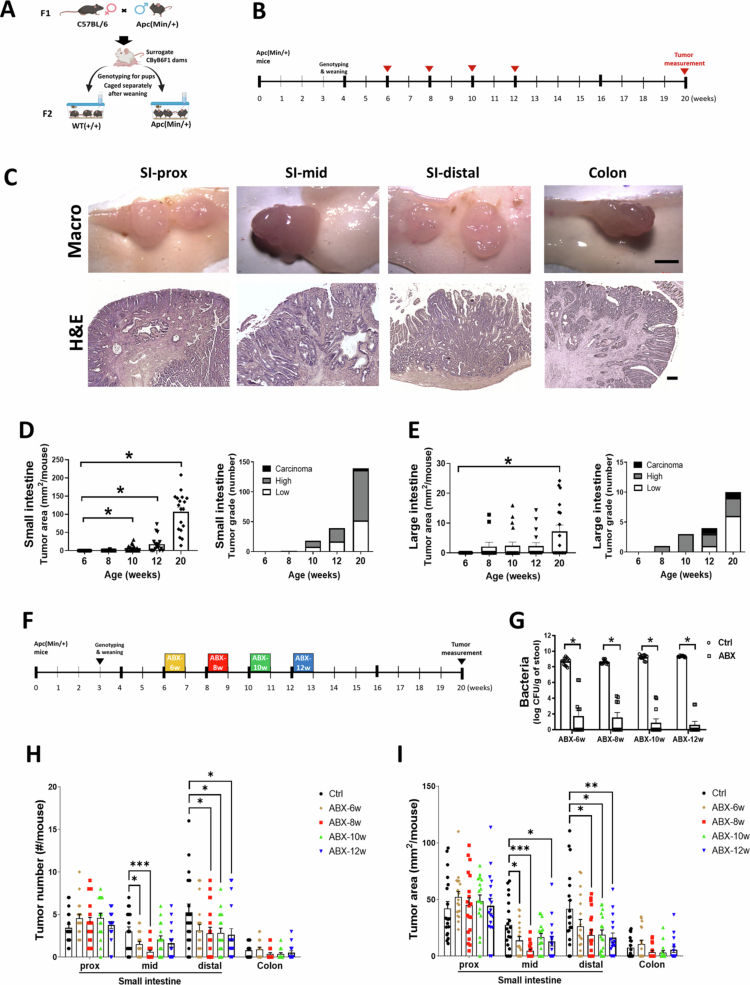
Gut microbiota depletion by antibiotic treatment reduced tumor burden in Apc(Min/+) mice. (A) Breeding strategy for generating mice with a single allele of *Apc* gene mutation. Embryos generated by *in vitro* fertilization of C57BL/6 oocytes with Apc(Min/+) sperm were injected into the womb of wild type (WT) CByB6F1 dams. The littermates were genotyped for cage separation after weaning. (B) Tumor formation in the small and large intestine of the Apc(Min/+) mice at 6, 8, 10, 12, and 20 weeks (w) of age. (C) Macroscopic inspection and tissue histology of small intestine (SI) and colon of 20-week-old Apc(Min/+) mice. Bar: 2 mm (macroscopic images) and 200 µm (histological images). (D and E) Tumors in the SI and colon of Apc(Min/+) mice at different ages. *p < 0.05 *vs.* 6w. *N* = 17–20/group. Left panel: tumor area. Right: tumor grade (low- and high-grade dysplasia and carcinoma). (F) Schematic diagram of Apc(Min/+) mice receiving one-week antibiotic (ABX) treatment at 6, 8, 10, or 12 weeks, and tumor measurement at 20 weeks of age. Age-matched control (Ctrl) group without ABX were used for comparison. (G) Depletion of fecal bacteria was confirmed after the ABX treatment. *p < 0.05 vs. Ctrl. *N* = 10/group. (H and I) A significant reduction in tumor number and area was observed in the mid- and distal-SI of Apc(Min/+) after ABX-8w treatment. *p < 0.05; **p < 0.01; ***p < 0.001 *vs.* Ctrl. *N* = 17–20/group. Statistical values were calculated by Kruskal–Wallis test followed by Dunn's test.

To investigate the role of microbiota in genetic cancers, tumor burden and microbiota composition were monitored longitudinally in littermates of Apc(Min/+) and WT(+/+) mice. The mouse embryos were generated by *in vitro* fertilization of C57BL/6 oocytes with Apc(Min/+) sperm, and were implanted into normogenic CByB6F1 female mice as surrogate dams. The littermates born to the surrogate dams were nursed in the same cage to ensure maternal transfer of eubiotic microbiota to the pups. The littermates were then weaned at 4 weeks after birth and genotyped for cage separation until 20 weeks of age (Figure 1A).

Since tumors develop in the small intestine (SI) and colon in FAP patients with *APC* mutations, the entire intestinal tract was examined in Apc(Min/+) mice at 6, 8, 10, 12, and 20 weeks of age (Figure 1B). No polyps were found in the SI segments of Apc(Min/+) mice by 6-8 weeks of age. Tumors with low-grade dysplasia were displayed in the proximal SI segment starting at 10 weeks, and with high-grade dysplasia at 12 weeks old. The middle and distal portions of the SI did not show tumors with dysplastic features until 12 weeks of age (Figure 1C–E). By 20 weeks of age, the proximal, middle, and distal SI segments of the Apc(Min/+) mice developed carcinoma (Figure 1C–E). As for the large intestine, dysplastic tumors were formed at 8 weeks, and carcinoma was found after 20 weeks of age (Figure 1C–E). In summary, SI tumors developed in all mice (100%), but colon tumors were observed in only half (50%) of the Apc(Min/+) mice by 20 weeks of age (Figure 1C–E). None of the WT(+/+) mice had tumors at any age.

Previous studies have shown an absence or low tumor burden in Apc(Min/+) when housed in germ-free conditions.^8^ ^,^ ^9^ ^,^ ^17^ To verify critical time points for host-microbiota interaction, Apc(Min/+) mice were treated with a one-week antibiotics (ABX) regimen at 6, 8, 10, or 12 weeks of age (Figure 1F). A significant reduction of fecal bacteria numbers was observed on the last day of each ABX session, confirming the bactericidal effects of broad-spectrum ABX cocktails (Figure 1G). Compared with the untreated control group, ABX treatment at 8 weeks of age (ABX-8w) significantly reduced tumor number and area in the middle-to-distal segments of the SI when examined at 20 weeks of age (Figure 1H–I). The other time points, i.e., ABX-6w, ABX-10w, and ABX-12w, had variable inhibitory effects on the middle-to-distal SI tumors, but none of the ABX regimens suppressed colon tumors or proximal SI tumors (Figure 1H–I). Overall, the data indicated that the timing of ABX treatment was an important determinant of tumor outcome, and confirmed that gut microbiota was involved in the tumorigenesis of Apc(Min/+) mice.

### Configuration of epithelial and fecal microbiota in Apc(Min/+) mice

**Figure 2.**
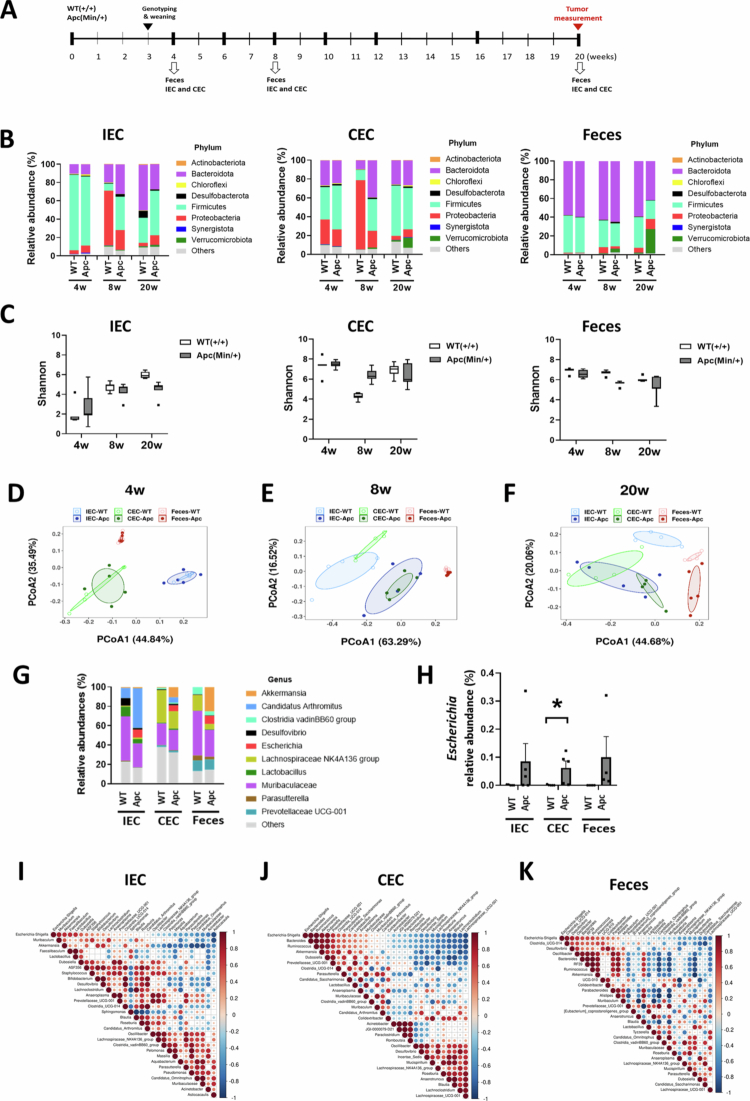
Longitudinal microbiota analysis unraveled early onset of epithelial dysbiosis prior to fecal dysbiosis in Apc(Min/+) mice. (A) Timeline for harvesting fecal and intestinal samples from Apc(Min/+) and WT(+/+) littermates at 4, 8, and 20 weeks (w) of age. The microbiota in fecal samples and epithelial cells of ileum (IEC) and colon (CEC) were analyzed using 16S rDNA sequencing. (B) Relative abundance of bacterial phyla of IEC, CEC, and fecal microbiota. (C) The alpha-diversity of IEC, CEC, and fecal microbiota. (D–F) PCoA plot of the relative abundance of OTUs in IEC, CEC, and fecal microbiota at 4w, 8w, and 20w. (G) Relative abundance of the top ten bacteria genera in epithelial and fecal microbiota at 20 weeks. *N* = 5/group. (H) A higher percentage of *Escherichia* genus in epithelial and fecal microbiota of Apc(Min/+) mice compared to WT(+/+) mice. *N* = 5/group. *p < 0.05 *vs.* WT(+/+). Statistical values calculated by Kruskal–Wallis test followed by Dunn's test. (I–K) Correlation heatmap showing multivariate analysis results expressed as Pearson's coefficients (from 1 to −1) among various bacterial genera in epithelial and fecal microbiota.

The microbiota composition in the epithelial and fecal compartments were compared between age-matched Apc(Min/+) and WT(+/+) littermates at 4, 8, and 20 weeks after birth (Figure 2A). The fecal samples and ileal (IEC) and colonic (CEC) epithelial cells were subjected to full-length 16S rDNA sequencing. The samples obtained at 4 weeks of age represent the phase when littermates are housed in the same cage and before tumor formation; those obtained at 8 weeks represent the tumor initiation phase in Apc(Min/+) mice after being cage-separated from their WT(+/+) littermates; those obtained at 20 weeks represent the carcinoma phase in Apc(Min/+) mice after being cage-separated from their WT(+/+) littermates (Figure 2A).

Based on the taxonomic analysis, eight major phyla in the epithelial microbiota and six major phyla in the fecal microbiota were identified in the Apc(Min/+) and WT(+/+) littermates (Figure 2B). While Bacteroidota and Firmicutes were the two dominant phyla that constituted 80-95% of the epithelial and fecal populations at 4 weeks of age, a significant increase in the relative abundance of Proteobacteria was noted at 8 weeks in the IEC, CEC, and fecal microbiota composition of both mouse genotypes (Figure 2B). For the alpha-diversity analysis, no statistical difference in microbiota richness was observed between Apc(Min/+) and WT(+/+) mice using the Shannon index at any age (Figure 2C). A trend of increased alpha-diversity in the IEC microbiota and a slight decrease in the CEC and fecal microbiota were noted at 8 weeks relative to 4 weeks of age in both mouse genotypes (Figure 2C).

The principal coordinate analysis (PCoA) revealed clustering of epithelial microbiota between Apc(Min/+) and WT(+/+) pups at 4 weeks old, as they are nursed by the surrogate dam in the same cage since birth (Figure 2D). The fecal microbiota of Apc(Min/+) and WT(+/+) pups were also clustered at this young age (Figure 2D). Notably, segregation of the epithelial microbiota was observed between Apc(Min/+) and WT(+/+) mice by 8 weeks of age, a time point at which the littermates were weaned and housed in separate cages according to their genotypes (Figure 2E). On the other hand, the fecal microbiota composition remains clustered for Apc(Min/+) and WT(+/+) mice at 8 weeks of age, despite being caged separately (Figure 2D). The data showed that while epithelial microbiota diverged as a result of host genotypes after being housed separately for one month, the fecal microbial population remained similar between Apc(Min/+) and WT(+/+) littermates. The fecal microbiota of the Apc(Min/+) mice did not diverge from that of WT(+/+) mice until 20 weeks of age (Figure 2F). Therefore, our results indicated that microbiota dysbiosis in the epithelial compartment (epithelial dysbiosis) was established earlier than that in the fecal samples (fecal dysbiosis). Moreover, the timing of epithelial dysbiosis was linked to the onset of tumor development, whereas fecal dysbiosis could be a consequence of epithelial dysbiosis or of cancer formation.

To pinpoint bacterial strains associated with tumorigenesis, we compared the relative abundances of the top 10 genera in each compartment using 16S rDNA sequencing. A significant increase in the relative abundance of *Escherichia* was observed in the CEC microbiota, and a trend of increase was also found in the IEC and fecal microbiota of Apc(Min/+) mice relative to WT(+/+) littermates (Figure 2G–H). Moreover, a positive correlation between *Escherichia* and *Akkermansia* was seen in all three microbiota compartments (i.e., IEC, CEC, and feces) of Apc(Min/+) mice (Figure 2I–K). *Roseburia*, *Lachnospiraceae* NK4A136, and UCG-001 groups showed negative correlations with *Escherichia* in the CEC and fecal microbiota compartments (Figure 2I–K). Moreover, *Lactobacillus* was positively correlated with *Escherichia* in the IEC and CEC microbiota, but negatively correlated with *Escherichia* in the fecal composition (Figure 2I–K). In summary, the data showed a higher abundance of *Escherichia* in the microbiota composition of Apc(Min/+) mice compared with those of WT(+/+) mice.

### Higher abundance of intraepithelial *E. coli* with invasive ability contributes to the distinct epithelial microbiota in Apc(Min/+) mice

**Figure 3.**
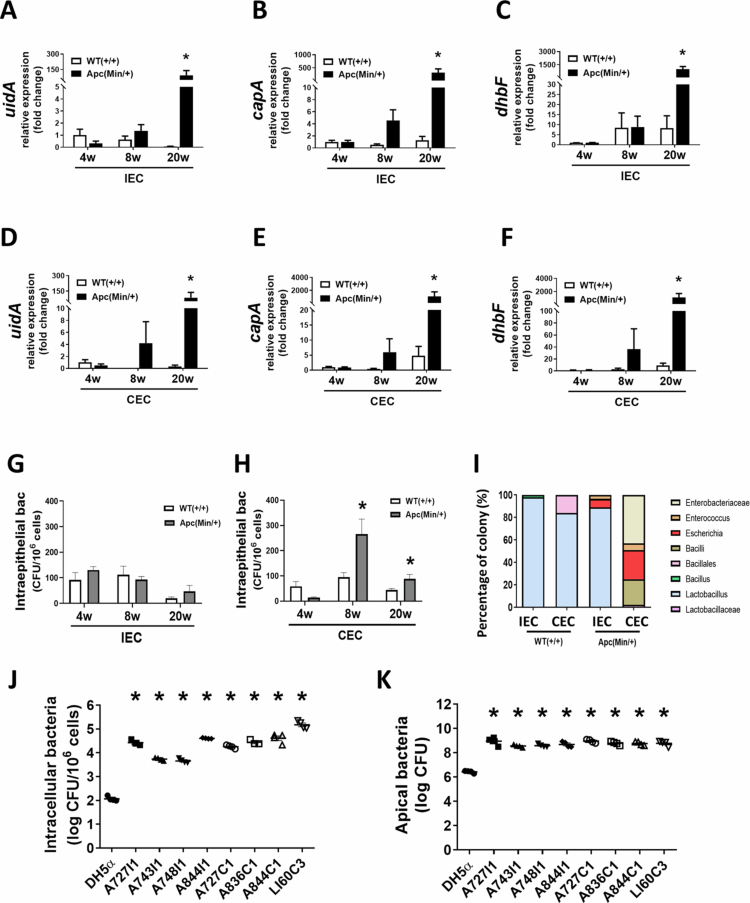
A high abundance of invasive *E. coli* in epithelial cells of Apc(Min/+) mice. Bacterial levels were quantified in the epithelial cells of ileum (IEC) and colon (CEC) samples of Apc(Min/+) and WT(+/+) mice by qPCR analysis. Primers targeting *uidA* (a universal *E. coli* gene), and targeting *capA* and *dhbF* (markers of an invasive *E. coli* LI60C3 strain, a tumorigenic pathobiont) were used. (A–C) Levels of *uidA*, *capA,* and *dhbF* genes in the IEC samples. (D–F) Levels of *uidA*, *capA,* and *dhbF* genes in the CEC samples. *N* = 10/group *p < 0.05 *vs.* WT(+/+). (G and H) Viable bacterial counts in epithelial cells using a gentamicin resistance assay. *N* = 10/group *p < 0.05 *vs.* WT(+/+). (I) Individual bacterial colonies were isolated for taxon characterization and shown as the percentage of bacterial strains in the intraepithelial population. (J and K) The intraepithelial *E. coli* strains were incubated with Caco-2 cells at MOI of 10 for 4 h to evaluate the intracellular and apical bacterial counts. The *E. coli* strains isolated from Apc(Min/+) mice, designated with the letter A in the nomenclature, exhibited a 1000-fold higher invasive ability than commensal DH5α. Comparable invasive ability was observed between LI60C3 and *E. coli* strains isolated from Apc(Min/+) mice. *p < 0.05 *vs.* DH5α. Statistical values calculated by ANOVA followed by Dunnett's test.

Bacterial gene expression was next quantified in intestinal epithelial cells isolated from mice at 4, 8, and 20 weeks of age. The levels of *uidA* (a universal *E. coli* gene), and *capA* and *dhbF* (genetic signatures of a tumorigenic *E. coli* strain LI60C3^14^ ^,^ ^15^ ^,^ ^20^) were measured in the IEC and CEC samples by using real-time PCR analysis (Figure 3A–F). Higher levels of invasive *E. coli* genetic signatures were observed in the epithelial samples of Apc(Min/+) mice relative to WT(+/+) mice by 20 weeks (Figure 3A–F).

To confirm the viability of the intraepithelial bacteria, freshly harvested epithelial cells were subjected to a gentamicin resistance assay, and the epithelial lysates were plated on agar to enumerate microbial colonies and characterize taxa. Although the intraepithelial bacterial counts of IEC samples were not different between Apc(Min/+) and WT(+/+) mice, a higher percentage of *Escherichia* was found in the IEC samples of Apc(Min/+) (Figure 3G and I). Elevated intraepithelial bacterial counts were found in the CEC samples of the 8- and 20-week-old Apc(Min/+) mice compared with age-matched WT(+/+) littermates (Figure 3H). The bacterial strains in the CEC samples of Apc(Min/+) mice were predominantly *Escherichia*, *Enterobacteriaceae, Enterococcus,* and *Bacilli* (Figure 3I). In contrast, only *Lactobacillus* or *Lactobacillaceae* were identified in the IEC and CEC samples of WT(+/+) mice (Figure 3I). The data indicated that a higher amount of live *Escherichia* is present in the epithelial compartment of Apc(Min/+) mice compared to WT(+/+) mice.

The intraepithelial *E. coli* strains isolated from Apc(Min/+) mice were named A748-I1, A727-I1, A743-I1, A844-I1, A727-C1, A775-C1, A837-C1, and A844-C1. In the given nomenclature, the letter “A” represents Apc(Min/+) mice, “I” signifies ileum, and “C” denotes colon. The invasive ability of each strain of intraepithelial *E. coli* was assessed by incubating the microbes with human Caco-2 cells at equal values of multiplicity-of-infection (MOI) of 10. The human Caco-2 cells exhibit a mutant *APC* gene harboring a single-nucleotide variant, similar to the genotypes of Apc(Min/+) mice.^49^ Our results showed that the intracellular bacterial counts in groups exposed to *E. coli* isolated from Apc(Min/+) mice were 1000-fold higher than those in groups exposed to the commensal *E. coli* DH5α (a sub-strain of K-12). Notably, the bacterial isolates from Apc(Min/+) mice exhibited an invasive ability comparable to that of LI60C3, a well-characterized tumorigenic *E. coli* pathobiont (Figure 3J). Moreover, the apical bacterial counts of *E. coli* strains from Apc(Min/+) mice were also significantly higher than those of commensal DH5α (Figure 3K). The results indicate that intraepithelial *E. coli* strains from Apc(Min/+) mice exhibited higher growth rates than commensal bacteria in epithelial cocultures. Collectively, the *E. coli* isolates from intestinal epithelial cells of Apc(Min/+) mice exhibited high invasiveness.

### Invasive *E. coli* promotes tumor growth and upregulates Hippo effector expression in Apc(Min/+) mice

**Figure 4.**
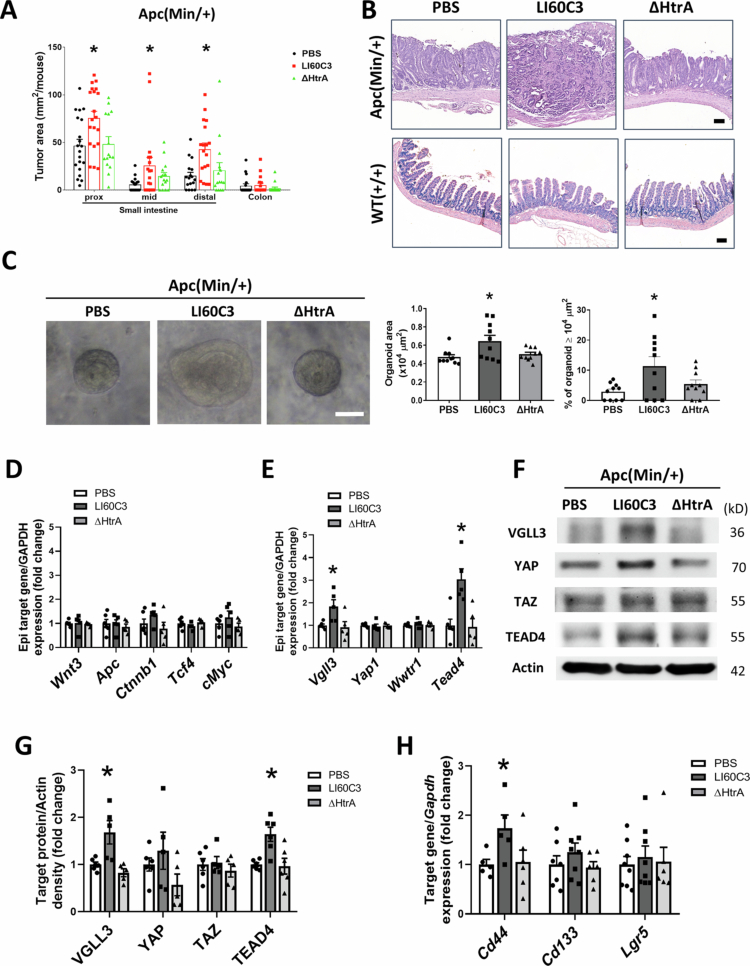
Inoculation of invasive *E. coli* increased tumor load and organoid sizes in Apc(Min/+) mice. Experimental pathobiont models were created by inoculating invasive *E. coli* LI60C3 into Apc(Min/+) and WT(+/+) mice to evaluate tumorigenic outcomes in hosts with different genetic backgrounds. (A) Apc(Min/+) mice inoculated with LI60C3 showed higher tumor burden compared to those with phosphate-buffered saline (PBS) or *htrA* gene-deleted bacteria (ΔHtrA). *p < 0.05 *vs.* PBS. *N* = 22–25/group. (B) Histological images of gut tissues in bacteria-infected mice. No sign of tumor was found in WT(+/+) mice inoculated with bacteria. Bar: 100 μm. (C) Intestinal tissues of Apc(Min/+) mice were harvested 2 d after the inoculation of LI60C3 to assess crypt-derived organoid sizes and epithelial gene expression. Representative images of intestinal organoid cultures, showing higher organoid sizes in the LI60C3 group. Bar: 50 mm. *N* = 10/group. *p < 0.05 *vs.* PBS. (D) Quantitative PCR analysis of Wnt/β-catenin pathway genes in mouse epithelial cells. (E) Quantitative PCR analysis of Hippo pathway genes in mouse epithelial cells. (F and G) Western blotting and densitometric analysis of Hippo effectors. (H) Quantitative PCR analysis of cancer stemness markers in tumor tissues of Apc(Min/+) mice, showing increased expression of *Cd44*, but not *Cd133* or *Lgr5*. *N* = 5–8/group. *p < 0.05 *vs.* PBS. Statistical values calculated by ANOVA followed by Dunnett's test.

To validate the tumorigenic effects of invasive bacteria, an experimental pathobiont model was employed by inoculating *E. coli* LI60C3 into Apc(Min/+) and WT(+/+) mice. A bioengineered bacterium lacking the stress-related *htrA* gene (ΔHtrA), which exhibited reduced intraepithelial survival, was also used for comparison with the isogenic LI60C3 strain.^14^ Inoculation with LI60C3 but not ΔHtrA increased intestinal tumor load in Apc(Min/+) mice compared with those administered a saline vehicle (Figure 4A). No sign of tumors was observed in the intestine of WT(+/+) mice after inoculation of LI60C3 (Figure 4B). Another mouse model in which chemical carcinogens were administered also showed a higher tumor load following inoculation with LI60C3 but not ΔHtrA (Suppl Figure 1). The data indicated that infection with invasive *E. coli* increased tumor burden in genetically susceptible mice but not in normogenic hosts.

The impact of invasive *E. coli* on cancer stemness and signaling molecules was examined in primary intestinal organoids and epithelial cells obtained from Apc(Min/+) mice. Increased organoid sizes were evident in the mouse group inoculated with LI60C3 compared to those with saline or ΔHtrA (Figure 4C). Quantitative PCR analysis showed no difference in the Wnt/beta-catenin pathways, such as *Wnt3, Apc, Ctnb1, Tcf7l2, and cMyc* gene expression, in the epithelial cells after LI60C3 inoculation (Figure 4D). In contrast, upregulated expression of the Hippo pathway-related cofactor *Vgll3* and the transcription factor *Tead4* genes, but not *Yap1* or *Wwtr1 (Taz)* gene, was observed in mouse epithelial cells after infection with LI60C3 but not with ΔHtrA (Figure 4E). Western blotting results also revealed higher levels of VGLL3 and TEAD4 proteins in epithelial samples from LI60C3-infected mice, as determined by densitometric analysis (Figures 4F, G). Moreover, significantly higher *Cd44,* but not *Cd133* or *Lgr5,* expression was noted in the tumor tissues of LI60C3-infected mice (Figure 4H).

### Invasive *E. coli* increases tumor clonogenicity in vitro

**Figure 5.**
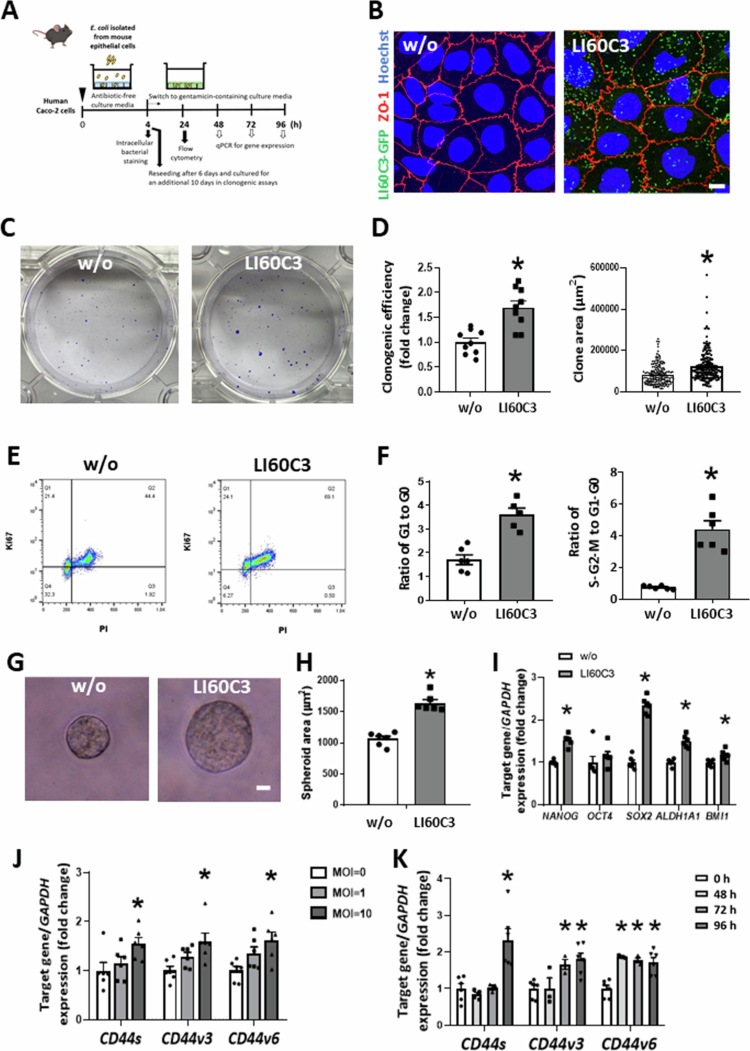
Increased tumor clonogenicity and active proliferation of Caco-2 cells after *E. coli* infection *in vitro*. (A) Schematic diagram of experimental timeline for bacteria-epithelial cocultures. Human Caco-2 cells were exposed to invasive *E. coli* LI60C3 for 4 h, then placed in gentamicin-containing culture media for a longer time point to assess clonogenicity and cell-cycle rates. (B) Representative immunofluorescent images showing the intracellular presence of LI60C3 in Caco-2 cells. The LI60C3 tagged with GFP (green) was merged with junctional ZO-1 staining (red) and cell nuclear staining (blue) in the images. Bar: 10 μm. (C) Representative tumorsphere images of bacteria-infected Caco-2 cells. (D) Higher clonogenicity of bacteria-infected Caco-2 cells. Clonogenic efficiency (left panel): Each dot represents the data of one well. *N* = 9 wells per group. Clone area (right panel): Each dot represents the data of one clone. *N* = 165–180 per group. *p < 0.05 *vs.* w/o. (E) Flow cytometric analysis of Caco-2 cell cycle rates. (F) Increased ratio of G1 to G0 phases (left panel) and ratio of S/G2-M to G1/G0 phases (right panel) after bacterial exposure. *N* = 6/group. *p < 0.05 *vs.* w/o. (G) Representative images of bacteria-infected tumorspheres. Bar: 10 μm. (H) Quantification of tumorsphere area. *N* = 6/group. *p < 0.05 *vs.* w/o. (I) Stem cell markers in bacteria-infected Caco-2 cells. *N* = 6/group. *p < 0.05 *vs.* w/o. (J and K) Levels of CD44 standard (s) and variant (v) isoforms in bacteria-infected Caco-2 cells at various MOIs and time points. *N* = 6/group. *p < 0.05 *vs.* 0. Statistical values calculated by ANOVA followed by Tukey's multiple comparison test or Student's *t* test when appropriate.

The self-renewal capacity of tumorspheres following bacterial infection was next evaluated in human Caco-2 epithelial cell cultures. The epithelial cells were exposed to invasive *E. coli* LI60C3 for 4 h in antibiotic-free culture media, and bacteria-infected cells were further incubated in gentamicin-containing media at extended time points to assess stemness and proliferation, using colonogenic assays, cell cycle cytometry, and qPCR analysis (Figure 5A). The intracellular presence of bacteria was confirmed by immunostaining (Figure 5B). An increase in the number and sizes of Caco-2 tumorspheres was observed in the bacteria-exposed group compared with uninfected controls (Figures 5C, D). For the cell cycle phases, a twofold higher G1-to-G0 ratio was observed in the bacteria-infected cells, suggesting an increased percentage of quiescent cells entering active proliferation. Moreover, a higher percentage of cells in the S and G2/M phases was noted following bacterial infection, suggesting accelerated cell cycle rates (Figure 5E and F). The flow cytometric data indicate that bacterial invasion shifts stem cells from a dormant state to division, and induces epithelial hyperproliferation. A significant increase in spheroid area was seen after bacterial challenge (Figure 5G and H). Several stem cell markers, such as NANOG, SOX2, ALDH1A1, and BMI1 was increased in the bacteria-infected Caco-2 cells (Figure 5I). Further, the CD44 standard isoform (CD44s) and the splicing variants 3 (v3) and 6 (v6) were increased in Caco-2 cells by 96 h after exposure to bacteria (Figure 5J). The timing of differential regulation of CD44 isoforms was also investigated. The mRNA levels of CD44v6 were elevated 48 h post-infection, whereas CD44v3 and CD44s levels increased at 72 and 96 h, respectively (Figure 5K). Overall, invasive *E. coli* promoted tumorsphere clonogenicity, shifting quiescent cells toward active proliferation, and accelerated cell cycle rates in association with higher CD44 expression in Caco-2 cells.

### Invasive *E. coli* promotes cancer stemness via upregulating VGLL3/TEAD4 pathways

**Figure 6.**
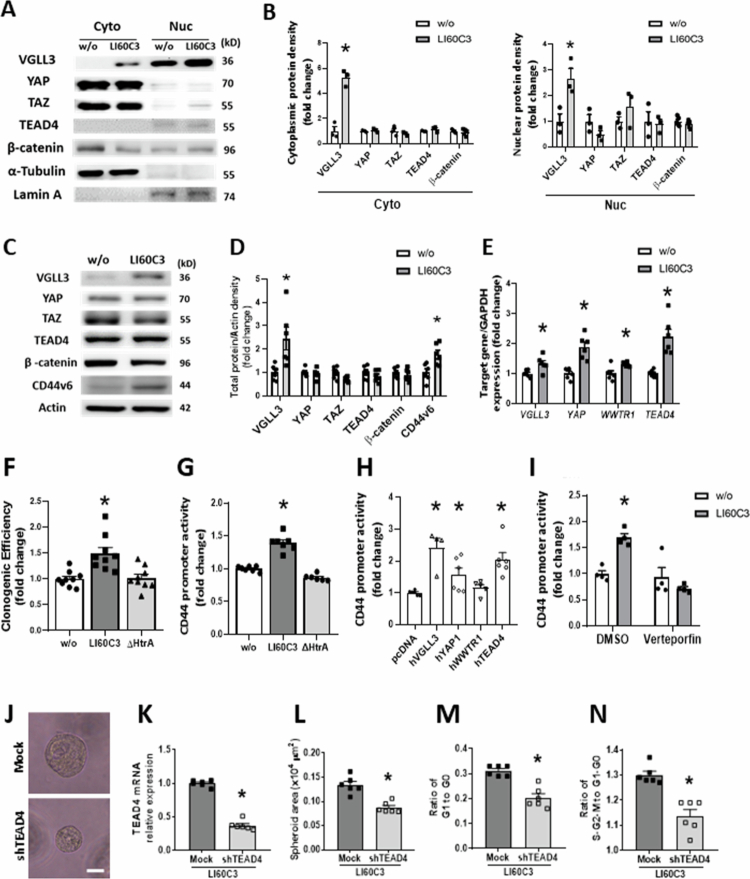
Bacterial infection fosters cancer stemness through upregulation of VGLL3/TEAD4-mediated CD44 transcriptional activity. (A and B) Western blots and densitometric analysis of the TEAD4 transcriptional cofactors, e.g., YAP1, TAZ, and VGLL3, in the cytoplasmic (Cyto) and nuclear (Nuc) fractions of bacteria-infected cells for 4 h. *N* = 6/group. *p < 0.05 *vs.* without bacteria (w/o). (C and D) Western blots showing increased VGLL3 and CD44v6 protein levels after exposure to invasive *E. coli* LI60C3 in Caco-2 cells for 24 h. *N* = 6/group. *p < 0.05 *vs.* w/o. (E) Quantitative PCR analysis of Hippo effectors in the epithelial cells after bacterial exposure for 96 h. *N* = 6/group. *p < 0.05 *vs.* w/o. (F and G) Exposure to LI60C3 but not ΔHtrA increased the clonogenic ability and CD44 promoter activity in Caco-2 cells. *N* = 6–9/group. (H) Overexpression of VGLL3, YAP1, and TEAD4 by plasmid transfection increased the luciferase-based CD44 promoter activity. *N* = 4–6/group. *p < 0.05 *vs.* pcDNA. (I) Exposure to invasive *E. coli* increased luciferase-based CD44 promoter activity in Caco-2 cells, which was prevented by verteporfin (an inhibitor of TEAD4 activation) but not DMSO vehicle. *N* = 4/group. *p < 0.05 *vs.* DMSO. (J) Representative images of bacteria-infected Caco-2 tumorspheres with mock treatment or lentivirus-mediated *TEAD4* gene silencing by shRNA. Bar: 10 μm. (K) Quantitative qPCR analysis to validate the knockdown of *TEAD4* gene. *N* = 6/group. (L, M, and N) Gene silencing of *TEAD4* reduced bacteria-infected tumorsphere area and epithelial cell cycle rates. *N* = 6/group. *p < 0.05 *vs.* mock. Statistical values were calculated by ANOVA followed by Tukey's multiple comparison test or Student's *t* test when appropriate.

To elucidate the signaling pathways involved in bacteria-induced cancer stemness, nuclear translocation and total protein levels of the Hippo pathway cofactors (e.g., VGLL3, YAP1, and TAZ) and transcriptional factor TEAD4 were assessed in the bacteria-infected Caco-2 cells. Western blotting results showed a significant increase in nuclear translocation of the VGLL3 protein, but not YAP, TAZ, or β-catenin molecules, in the Caco-2 cells after bacterial exposure for 4 h (Figure 6A, B). An increase in total VGLL3 protein was associated with higher CD44v6 levels in bacteria-infected cells after 24 h (Figure 6C, D). In contrast, the total protein levels of YAP, TAZ, or TEAD4 were not increased in the bacteria-infected cells after 24 h (Figure 6C, D). Notably, the transcript levels of *VGLL3, YAP, WWTR1 (TAZ),* and *TEAD4* were elevated in bacteria-infected cells by 96 h. The findings suggest that invasive *E. coli* primarily triggered nuclear shuttle of VGLL3 and caused a secondary upregulation of the Hippo effectors at later time frames (Figure 6E).

We next compared the tumorsphere formation and CD44 promoter activity induced by LI60C3 and ΔHtrA at equal MOIs. Unlike LI60C3 infection, ΔHtrA failed to increase the clonogenicity of Caco-2 cells nor upregulate CD44 promoter activity (Figure 6F, G). Overexpression of TEAD4 or its cofactors via plasmid transfection increased CD44 promoter activity (Figure 6H), confirming that Hippo effectors act as transcriptional regulators to promote CD44 expression as a target gene. Pretreatment with verteporfin (an inhibitor of TEAD4 activation) significantly reduced the increased clone numbers in Caco-2 cells induced by bacteria (Figure 6I). Further, gene silencing of *TEAD4* led to decreases in bacteria-induced spheroid area and epithelial cell cycle rates (Figure 6J–N).

### Positive correlation between invasive *E. coli* and Hippo effectors in human CRC

**Figure 7.**
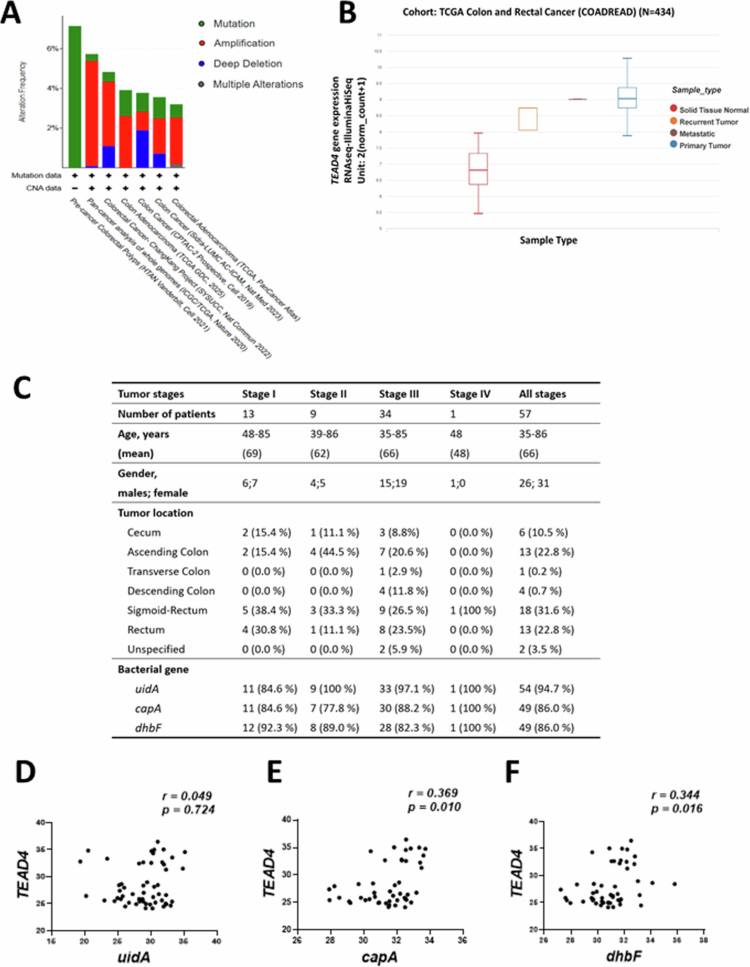
A positive correlation between invasive *E. coli* and TEAD4 expression in human CRC specimens. A bioinformatics approach using The Cancer Genome Atlas (TCGA) datasets, combined with qPCR analysis, was performed to validate the correlation in clinical samples. (A) Large-scale bioinformatics analysis using cBioPortal showed that approximately 3%–7% of CRC patients exhibited *TEAD4* gene alterations, including mutations and copy number alterations (CNAs), based on TCGA datasets comprising 2591 patients across six studies. (B) Increased expression of the *TEAD4* gene was observed in primary tumors compared with normal tissues, based on TCGA Colon and Rectal Cancer (COADREAD) datasets comprising 434 samples generated by the Xena program. Statistical significance (*p* < 0.001) among groups was established by ANOVA. (C) Characteristics of CRC patients by tumor stages and locations. Surgical specimens were collected from a cohort of 57 patients, including those with tumor stages I, II, III, and IV. The universal *E. coli uidA* gene and the LI60C3-specific *capA and dhbF* genes were detected in more than 86% of the CRC patients. (D) No correlation was observed between universal *E. coli uidA* and TEAD4 gene expression. (E and F) LI60C3-specific *capA* and *dhbF* levels correlated positively with *TEAD4* expression in human CRC. Each dot in the scatter plots represents data from one patient.

To further assess the translational relevance, *in silico* analysis of *TEAD4* gene alterations in human CRC datasets was performed. Using The Cancer Genome Atlas (TCGA) datasets from 2591 patients across 6 studies, approximately 3%–7% of CRC patients exhibited *TEAD4* gene mutations and copy number alterations, as analyzed using cBioPortal (Figure 7A). It is noteworthy that 7% of pre-cancerous colorectal polyps exhibited *TEAD4* gene mutation, and 3%–6% of CRC samples showed *TEAD4* gene amplification. Moreover, higher *TEAD4* gene expression was observed in primary tumors than in normal tissues, based on the TCGA Colon and Rectal Cancer (COADREAD) datasets comprising 434 samples generated by the Xena program (Figure 7B).

The TCGA datasets are tailored to reveal host genomic and transcriptomic alterations, but lack information on the microbiota. Therefore, human CRC specimens were collected to evaluate the relationship between *E. coli* LI60C3 levels and *TEAD4* gene expression. Surgical specimens from a cohort of 57 patients, including those with tumors in stage I (*N* = 13), stage II (*N* = 9), stage III (*N* = 34), and stage IV (*N* = 1), were assessed (Figure 7C). Our pilot studies showed that the LI60C3-specific capA and dhbF genes were detected in only about 25% of cDNA samples reverse-transcribed from RNA. In contrast, these bacterial markers could be identified in more than 80% of genomic DNA samples extracted from human CRC. To obtain sufficient numbers to calculate the Pearson correlation coefficient (r) between two variables, we assessed bacterial levels in genomic DNA samples and quantified human gene expression in RNA/cDNA samples from the same cohort. The percentages of CRC patients positive for the universal *E. coli uidA* gene and LI60C3-specific *capA* and *dhbF* genes, using genomic DNA samples, are 94.7%, 86.0%, and 86.0%, respectively (Figure 7D). As the *uidA* gene is present in all *E. coli* strains without specifying pathobionts, no association was found between *TEAD4* expression and *uidA* levels (Figure 7E). Notably, the LI60C3 genetic signatures (i.e., *capA* and *dhbF*) correlated positively with human *TEAD4* expression in tumor specimens, with statistical significance (Figure 7F, G).

## Discussion

Our research indicates that tumor development in Apc(Min/+) mice results not only from mutations in host genes but also from microbiota dysbiosis. Eubiotic commensal bacteria acquired in early life converted into pathobionts in adult Apc(Min/+) mice, and the host genetics-driven expansion of invasive *E. coli* was responsible for enhanced tumor clonogenicity. The invasive *E. coli* directly promotes cancer stemness through a VGLL3/TEAD4/CD44 axis, independent of immune cell activation. Moreover, the genetic signatures of invasive *E. coli* were positively correlated with TEAD4 expression in human CRC specimens, suggesting clinical relevance in tumorigenesis.

Our study showed that antibiotic treatment reduced tumor burden in the mid and distal SI but not in the proximal SI and colon of Apc(Min/+) mice, supporting the involvement of microbiota in genetics-associated intestinal tumorigenesis. The regional differences in ABX treatment efficacy likely reflect the complex interplay between microbiota composition, immune responses, and local tissue environments across different segments of the intestine. The microbiome composition in Apc(Min/+) and WT(+/+) littermates nursed by the WT dams was monitored longitudinally through adulthood to evaluate how genetic factors shape the gut ecosystem. The segregation of epithelial microbiota occurred earlier than the divergence of fecal microbiota between Apc(Min/+) and WT(+/+) littermates, suggesting that the onset of epithelial dysbiosis preceded tumor formation in the genetic mutant mice. This also suggests a localized disruption of the epithelial microbiome early in disease progression, even before widespread alterations are seen in the fecal microbial landscape. Elevated intraepithelial bacterial counts, particularly *E. coli*, were observed in Apc(Min/+) mice. This could be explained by previous observations that epithelial cancers driven by *Apc* allelic loss also exhibited defective expression of junctional proteins, higher luminal-to-serosal macromolecular flux, and increased permeability to bacteria.^10-13^ Consistent with this finding, microbiota dysbiosis and the emergence of invasive *E. coli* were reported in genetically modified mice with epithelial barrier defects, despite inheriting eubiotic commensals at birth.^20^ ^,^ ^21^ ^,^ ^50-52^ Notably, the intraepithelial *E. coli* isolated from Apc(Min/+) mice exhibited greater invasive capacity than commensal bacterial strains, suggesting pathobiont traits.

A direct role of invasive bacteria on cancer stemness was evaluated *in vitro* using primary organoids and epithelial cell cultures. The invasive *E. coli* LI60C3 not only increased mouse tumor burden but also enhanced tumorsphere formation *in vitro*, which provided clear evidence that cancer stemness triggered by invasive *E. coli* is independent of immune cell activation. This feature contrasts with that of other tumor-associated pathobionts, such as *Bacteroides fragilis* and *Fusobacterium nucleatum*, which promote cancer development by modulating the immune response through induction of Th17 responses and inhibition of NK cell cytotoxicity.^53^ ^,^ ^54^ The invasive *E. coli* strain LI60C3 was initially isolated from mouse intestinal epithelial cells, in which colibactin (clb) or polyketide synthase (pks) were absent from the genome, as revealed by whole-genome sequencing.^14^ ^,^ ^15^ The findings suggest that, besides genotoxicity, there are alternative tumorigenic mechanisms conferred by LI60C3. We previously demonstrated that invasive *E. coli* suppressed host autophagy to benefit their own survival within cells, stimulated free radical-dependent epithelial hyperproliferation, and induced epithelial circadian-driven inflammation.^14^ ^,^ ^20^ In this study, *E. coli* pathobionts triggered Hippo signaling pathways and downstream epigenetic changes associated with cancer stemness in epithelial cells, in addition to the existing *Apc* gene mutation. This may explain why Apc(Min/+) mice raised in germ-free conditions do not develop neoplasia or exhibit a low tumor burden, despite dysregulated Wnt/β-catenin signaling. A recent study indicated that *E. coli* genotoxins are associated with *Apc* gene inactivation and early-onset CRC that occurs before the age of fifty.^55^ Collectively, the emergence of intestinal pathobionts and dysbiosis-induced cancer stemness may contribute to the rising prevalence of early-onset cancers.

We identified that *E. coli* infection triggered nuclear shuttling of VGLL3, serving as a coactivator of TEAD4-mediated CD44 promoter activity. Hippo effectors VGLL3 and TEAD4 are novel cancer stemness-related prognostic signatures related to chemoresistance in colon adenocarcinoma.^56^ ^,^ ^57^ Although nuclear translocation of YAP/TAZ was not observed in bacteria-infected cells, mRNA levels of these molecules increased at later time points, suggesting a possible role for these Hippo effectors in chronic tumor growth. Hippo pathways integrate a multitude of inputs, including mechanotransduction and cytoskeletal signals. A separate line of evidence has shown that Hippo effectors are involved in the host defense mechanism against intracellular pathogens, such as *Salmonella enterica*, *Staphylococcus aureus, Legionella pneumophila,* and *Pseudomonas aeruginosa,* in epithelial cells and phagocytes.^58-61^ These invasive pathogens are known to stimulate actin filament polymerization to facilitate cell penetration, a phenomenon also observed with invasive *E. coli*. Consistent with the findings, we observed that invasive *E. coli* exploited epithelial Hippo-TEAD4 pathways to enhance cancer stemness.

Accumulating evidence has implicated the two signaling pathways, i.e., Wnt and Hippo, closely cooperating to regulate the self-renewal and regeneration of intestinal stem cells. While it is well known that CD44 is a target gene downstream of the Wnt/β-catenin/TCF signals,^62^ ^,^ ^63^ we demonstrate that TEAD4 activity also drives the transcription of this cancer stemness marker.^33^ The microbial activation of Hippo signaling may act independently or synergistically with Wnt-driven β-catenin nuclear translocation to further amplify CD44 expression. Whether the upregulation of standard and splicing variants of CD44 is separately controlled by the Wnt/β-catenin and VGLL3/TEAD4 signals warrants further investigation. An alternative explanation is that the *Apc* gene mutation that sustains the Wnt/β-catenin pathway primes epithelial cells for enhanced responsiveness to invasive *E. coli*, perhaps by upregulating Hippo pathway molecules. Previous studies have demonstrated that the Wnt pathway leads to the release of YAP/TAZ from the β-catenin destruction complex, thereby facilitating their translocation to the nucleus for target gene expression.^64^ Conversely, YAP mediates the activation of the Wnt pathway. Nuclear YAP can directly bind to β-catenin/TCF, forming a YAP/β-catenin/TCF complex, thus activating the Wnt/β-catenin pathway.^65^ Taken together, a crosstalk between the Wnt and Hippo pathways may underlie the integrated mechanistic framework linking host genetics, microbial dynamics, and cancer stemness.

Lastly, higher *TEAD4* gene expression was validated in human CRC samples using large-scale bioinformatics. The TCGA datasets are tailored to reveal host genomic alterations, but lack information on the microbiota. To circumvent this challenge, we extracted genomic DNA and RNA samples from CRC specimens to assess the relationship between microbial and host gene expression. The clinical specimens showed a positive correlation between tumorigenic *E. coli* signatures and TEAD4 levels, supporting an association between the Hippo pathway and the presence of *E. coli* pathobionts. Results from clinical samples suggest that genetic signatures of the tumorigenic *E. coli* may serve as predictors of CRC progression. Epithelial microbiota profiling could serve as an early biomarker for individuals at higher risk of CRC, including those with colitis-associated and hereditary cancers, before noticeable changes in the fecal microbiota and overt tumor formation. Collectively, microbiome-targeted approaches, such as bacteriophage therapy, next-generation probiotics, or small molecules that specifically inhibit invasive *E. coli* virulence, could serve as potential anticancer strategies.^66^

In conclusion, host genetics-driven expansion of invasive *E. coli* exacerbated tumorigenesis through induction of cancer stemness. The invasive pathobionts increased tumor clonogenicity associated with CD44 upregulation *via* a VGLL3/TEAD4 axis. Tumorigenic *E. coli* genetic signatures may serve as biomarkers of CRC progression. Understanding how microbial dynamics interact with epithelial cell behavior is crucial for uncovering potential therapeutic targets in cancer prevention and treatment. Our data support the use of bacterial precision medicine as an alternative strategy for managing CRC, even in the hereditary subset.

## Supplementary Material

Supplementary Material Supplementary Figures

Supplementary Material Suppl_Tables_primer_pairs_Gut_Microbes clean.docx

## Data Availability

The bacterial 16S rDNA sequencing data were submitted to the GEO database (GSE245618).

## Abbreviations

ABX: antibiotics
APC: adenomatous polyposis coli
CRC: colorectal carcinoma
FAP: familial adenomatous polyposis
LDA: linear discriminant analysis
MOI: multiplicity-of-infection
PCR: polymerase chain reaction
PCoA: principal coordinate analysis
TAZ: transcriptional coactivator with PDZ-binding motif
TEAD4: TEA Domain Transcription Factor 4
VGLL3: Vestigial-like family member 3
YAP: yes-associated protein
