# Supplementary material for: Emergence of invasive Escherichia coli pathobionts in gut microbiome promotes cancer stemness via targeting Hippo pathways
Source: Gut Microbes. 2026 Jun 30;18(1):2694795. doi: 10.1080/19490976.2026.2694795 (PMC13327355; doi:10.1080/19490976.2026.2694795)

**Supplementary Figures**

**Suppl Fig. 1 Invasive E. coli LI60C3 promotes tumor growth in mutagen-induced colon cancer mouse model.** Mice administered azoxymethane and dextran sodium sulfate (AOM/DSS) were inoculated with PBS, LI60C3, or ΔHtrA. A larger tumor area was observed in the LI60C3 group compared to the PBS and ΔHtrA groups. **P*<0.05 *vs.* PBS. N=8/group.


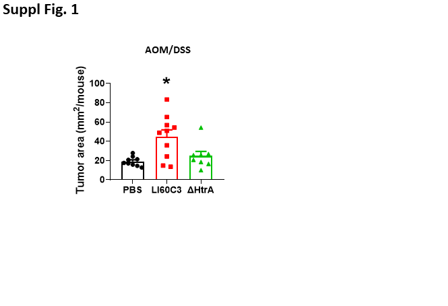

Supplement: Supplementary Material — Supplementary Figures [file KGMI_A_2694795_SM5208.docx]
