# Supplementary material for: Emergence of invasive Escherichia coli pathobionts in gut microbiome promotes cancer stemness via targeting Hippo pathways
Source: Gut Microbes. 2026 Jun 30;18(1):2694795. doi: 10.1080/19490976.2026.2694795 (PMC13327355; doi:10.1080/19490976.2026.2694795)
Supplement: Supplementary Material — Suppl_Tables_primer_pairs_Gut_Microbes clean.docx [file KGMI_A_2694795_SM9571.docx]

**Suppl Table 1 Primer pairs for mouse genes in quantitative PCR analysis**

| **Gene** | **Oligonucleotide sequence (5'→3')** | **Size (bp)** | **Tm (°C)** |
| --- | --- | --- | --- |
| *Cd44* | F: GCACTGTGACTCATGGATCC  R: TTCTGGAATCTGAGGTCTCC | 104 | 60 |
| *Cd44s* | F: AAGAGCACCCCAGAAAGCTAC  R: TCTCGATCTCTGGTGGCCAA | 54 | 60 |
| *Cd44v3* | F: ACCAGAGATCGAGACTCATCCA  R: GATCCATGAGTCACAGTGCGG | 56 | 60 |
| *Cd44v6* | F: CCTTGGCCACCACTCCTAATAG  R: CAGTTGTCCCTTCTGTCACATG | 132 | 60 |
| *Cd133* | F: CCTCTGGTGGGCTGCTTCT  R: CTCCGCCGCATTTGTTG | 55 | 60 |
| *Lgr5* | F: GCGCCTAGATGCCAACCA  R: GAGTGCAGGCCGCTGAAA | 60 | 60 |
| *Vgll3* | F: CCCAGACGACTTCCTCAACAG  R: GGGTCTCTTTGGCTGATGGT | 56 | 60 |
| *Yap* | F: TGCTCCCCAGTGCGACA  R: TGAGGTAGCCGTGGTGACTGT | 61 | 60 |
| *Wwtr1*  *(Taz)* | F: GACCCGCTTCCCTGATTT  R: AAGTCCCGAGGTCAACATTTGT | 59 | 60 |
| *Tead4* | F: ACGCGCACAAGGAAGCA  R: TTTTCGACGGGCAAGCA | 54 | 60 |
| *Wnt3* | F: ACCGAGAGGGACCTGGTCTAC  R: CTGGGTTGGGCTCACAAAA | 58 | 60 |
| *Apc* | F: CTGCCAGAGTGACACCTTTTAATTAC  R: GACGGCCGGGCTGAA | 76 | 60 |
| *Ctnnb1* | F: TGAATGGGAGCAAGGCTTTT  R: CTGCCCGTCAATATCAGCTACTT | 64 | 60 |
| *Tcf7l2* | F: AGGTGGTGGCCGAATGC  R: CCTGCGCCCGAGAATCT | 62 | 60 |
| *cMyc* | F: GTCTTTCCCTACCCGCTCAAC  R: GTGGAATCGGACGAGGTACAG | 62 | 60 |
| *Gapdh* | F: CAT GGC CTT CCG TGT TCC TA  R: GCG GCA CGT CAG ATC CA | 60 | 60 |

**Footnote:** The primer sequences were designed based on the gene sequence in the NCBI database. The annealing temperature (*Tm*) for all primer pairs is set to 60°C.

**Suppl Table 2 Primer pairs for human genes in quantitative PCR analysis**

| **Gene** | **Oligonucleotide sequence (5'→3')** | **Size (bp)** | **Tm (°C)** |
| --- | --- | --- | --- |
| *CD44* | F: GGACACCCCAAATTCCAGAA  R: AATCAAAGCCAAGGCCAAGA |  | 60 |
| *CD44s* | F: ACGAAGACAGTCCCTGGATCA  R: AATGTGTCTTGGTCTCTGGTAGCA | 67 | 60 |
| *CD44v3* | F: CAGGCTGGGAGCCAAATG  R: AATGCCTGATCCAGAAAACTGA | 68 | 60 |
| *CD44v6* | F: GAAACAGCTACCCAGAAGGAACA  R: TGTTTGGCGATATCCCTCATG | 61 | 60 |
| *CD133* | F: GCTGTGCGGGAACTCCTTT  R: AGCCTTAGGAGCATCTGTGGAT | 58 | 60 |
| *LGR5* | F: CCCTTCATTCAGTGCAGTGTTC  R: GCAGGTGTTCACAGGGTTTGA | 60 | 60 |
| *VGLL3* | F: CTCCCCCTGCTGTGTCTGA  R: ATGGGCTCACCTGAGATGTCA | 58 | 60 |
| *YAP* | F: CCAGTGCAGCAGAATATGATGAA  R: GGCTTGTTCCCATCCATCAG | 59 | 60 |
| *WWTR1*  *(TAZ)* | F: CAGAAACTGCGGCTTCAGAGA  R: CCTCTTGGCGCATTCGAA | 61 | 60 |
| *TEAD4* | F: TGTGGCAGGCGCAAAAT  R: GCTCGTTCCGACCATACATCTT | 58 | 60 |
| *WNT3* | F: GCCTGGTCCCCAAGCAA  R: GCTGGGCATGATCTCGATGT | 56 | 60 |
| *APC* | F: CACCTCAAGTTCCAACCACATTT  R: CATGATTAGAACCCACTCGATTTG | 103 | 60 |
| *CTNNB1* | F: CCCTGGTGAAAATGCTTGGT  R: TGGAGAGTTGTAATGGCATAAAACA | 64 | 60 |
| *TCF7L4* | F: ACACACGACGGGCATTCC  R: GGACGATTCCTGTTTGACTGTTG | 61 | 60 |
| *cMYC* | F: GAGGCGAACACACAACGTCTT  R: CACGCAGGGCAAAAAAGCT | 71 | 60 |
| *GAPDH* | F: AACGGGAAGCTTGTCATCAATGGAA  R: GCATCAGCAGAGGGGGCAGAG | 63 | 60 |

**Footnote:** The primer sequences were designed based on the gene sequence in the NCBI database. The annealing temperature (*Tm*) for all primer pairs is set to 60°C.

**Suppl Table 3 Primer pairs of quantitative PCR for bacterial genes**

| **Gene** | **Oligonucleotide sequence (5'-3')** | **Size (bp)** | **Ref.** |
| --- | --- | --- | --- |
| *uidA* | *F*: CGG AAG CAA CGC GTA AAC TC  *R*: TGA GCG TCG CAG AAC ATT ACA | 70 | ^1^ |
| *htrA* | *F:* CCA TTG CGA TAT ACC CAA ACT T  *R:* CTG GTT TCC AAG AGG GTG AT | 130 | ^2^ |
| *capA* | *F*: TGT TGG AAA ACG CGG TGA TA  *R*: CTT CGA AGT GTA AAA GCC ATG TTT AT | 78 | This study |
| *dhbF* | *F*: CGT GGC CGA GTT GAT TTT ACA  *R*: CCG TTG ATT AGC GCA GTT TTC | 63 | This study |
| *16S rRNA* | *F*: CCT ACG GGA GGC AGC AG  *R*: ATT ACC GCG GCT GCT GG | 194 | ^3^ |

**Footnote:** All real-time PCR primers were designed in this study, except those cited from references. The annealing temperature (*Tm*) for all primer pairs is set to 60°C.

**References of Supplemental Tables**

1. Walker DI, McQuillan J, Taiwo M, et al. A highly specific Escherichia coli qPCR and its comparison with existing methods for environmental waters. Water Res 2017;126:101-110.

2. Bui XT, Qvortrup K, Wolff A, et al. Effect of environmental stress factors on the uptake and survival of Campylobacter jejuni in Acanthamoeba castellanii. BMC Microbiol 2012;12:232.

3. Watanabe K, Kodama Y, Harayama S. Design and evaluation of PCR primers to amplify bacterial 16S ribosomal DNA fragments used for community fingerprinting. J Microbiol Methods 2001;44:253-62.
